# Progress in Reversing the HIV Epidemic through Intensified Access to Antiretroviral Therapy: Results from a Nationally Representative Population-Based Survey in Kenya, 2012

**DOI:** 10.1371/journal.pone.0148068

**Published:** 2016-03-01

**Authors:** Andrea A. Kim, Irene Mukui, Lucy N’gan’ga, Abraham Katana, Dan Koros, Joyce Wamicwe, Kevin M. De Cock, KAIS Study Group

**Affiliations:** 1 US Centers for Disease Control and Prevention, Division of Global HIV and Tuberculosis, Nairobi, Kenya; 2 Ministry of Health, National AIDS and STI Control Programme, Nairobi, Kenya; British Columbia Centre for Excellence in HIV/AIDS, CANADA

## Abstract

**Background:**

In 2014, the Joint United Nations Programme on HIV/AIDS (UNAIDS) called for 90% of persons living with HIV (PLHIV) to know their status, 90% of these to be on antiretroviral therapy (ART), and 90% of these to be virally suppressed by 2020 (90-90-90). It is not clear whether planned ART scale-up in countries whose eligibility criteria for ART initiation are based on recommendations from the 2013 World Health Organization treatment guidelines will be sufficient to meet UNAIDS' new global targets.

**Materials and Methods:**

Using data from a nationally representative population-based household survey of persons in Kenya we compared coverage and unmet need associated with HIV diagnosis, ART, and viral suppression among PLHIV aged 15–64 years in 2012 based on criteria outlined in the 2014 national ART guidelines and UNAIDS’ 90-90-90 goals. Estimates were weighted to account for sampling probability and nonresponse.

**Results:**

Eight in ten PLHIV aged 15–64 years needed ART based on treatment eligibility. Need for treatment based on the national treatment policy was 97.4% of treatment need based on UNAIDS’ 90-90-90 goals, requiring an excess of 24,000 PLHIV to access treatment beyond those eligible for ART to achieve UNAIDS’ 90-90-90 treatment target. The gap in treatment coverage was high, ranging from 43.1% nationally to 52.3% in Nyanza among treatment-eligible PLHIV and 44.6% nationally to 52.4% in Nyanza among all PLHIV.

**Conclusion:**

Maintaining the current pace of ART scale-up in Kenya will result in thousands of PLHIV unreached, many with high viral load and at-risk of transmitting infection to others. Careful strategies for reaching 90-90-90 will be instrumental in determining whether intensified access to treatment can be achieved to reach all who require ART.

## Introduction

With the potential of universal HIV treatment in sub-Saharan Africa (SSA), we may be able to reverse the tide in the HIV pandemic. Not only does successful treatment prevent HIV-related illness and death, it has substantial prevention benefits by reducing population-level HIV incidence through suppression of individuals’ viral load. Since the early 2000s impressive strides have been made in scaling up HIV treatment globally, resulting in almost 12.9 million people that have accessed HIV treatment by 2014 and 7.6 million deaths averted, including 4.8 million in SSA [1]. Despite this, high levels of new and undiagnosed infections persist, with 2.1 million new infections occurring globally and over 50% of persons living with HIV unaware of their infection in 2013 [1].

In 2013, the World Health Organization (WHO) released normative guidance on the use of ART to treat and prevent HIV infection [2]. These guidelines recommended earlier treatment of persons living with HIV (PLHIV) by increasing immunologic eligibility criteria for children aged 5–17 years and adults aged 18 years and older from CD4 ≤ 350 cells/mm^3^ to CD4 ≤ 500 cells/mm^3^. Also recommended was immediate ART for HIV-infected adults with active tuberculosis disease, chronic Hepatitis B virus infection, or advanced clinical disease (WHO stage III/IV); HIV-infected persons in serodiscordant relationships; HIV-infected pregnant and breastfeeding women; and HIV-infected children younger than aged five years, all irrespective of CD4+ T-cell count level.

New global targets from the Joint United Nations Programme on HIV/AIDS (UNAIDS) were released in 2014 that called for further impact on the HIV epidemic by treating as early as possible and monitoring progress through harmonized targets [3]. These targets were 90% of PLHIV to know their HIV status, 90% of PLHIV who know their status to receive life-saving antiretroviral therapy (ART), and 90% of PLHIV on ART to have suppressed viral load by 2020 to end the HIV pandemic by 2030 (90-90-90). Subsequently in 2015, in further support of early treatment, the WHO released updated guidelines for ART, recommending immediate treatment for all persons diagnosed, regardless of CD4+ T-cell count level [4].

SSA carries the highest burden of HIV disease, accounting for nearly 70% of PLHIV globally [1]. Reversing the HIV pandemic requires drastic reductions in HIV transmission and new HIV infections in high burden countries in SSA through sustained, community-wide suppression of the virus. Kenya, a SSA country in Eastern Africa, has the fourth largest HIV epidemic in the world [1]. By year-end 2012, approximately 1.2 million adults were living with HIV/AIDS in the country and 100,000 new HIV infections were occurring annually [5]. The region of Nyanza in Western Kenya carries the heaviest burden of HIV in the country, with 15% of the adult population afflicted with the disease [5].

In June 2014, Kenya revised its national guidelines for HIV treatment in alignment with the 2013 WHO guidelines on ART eligibility [6]. Concurrently, the country launched a new five-year strategic plan to guide the national HIV response from 2014 to 2019 which included strategies for achieving UNAIDS’ 90-90-90 targets for HIV diagnosis, ART, and viral suppression [7]. By year-end 2014, ART coverage among adult PLHIV under Kenya’s new treatment guidelines was estimated to be 57% [8]. As Kenya implements its new strategic vision for HIV control it is not clear whether planned treatment scale-up based on current national treatment guidelines will be sufficient to meet the 90-90-90 goals.

High-quality population-based data on HIV diagnosis, treatment status, and viral suppression among HIV-infected adults can be used to quantify coverage and unmet need based on national eligibility criteria and UNAIDS’ metrics for treatment. The Kenya AIDS Indicator Survey (KAIS) is a national population-based HIV survey conducted every five years to monitor trends in HIV prevalence, incidence, behavior, and coverage of HIV programs [9]. We used data from the second KAIS (KAIS 2012) to compare coverage and unmet need associated with diagnosis, ART, and viral suppression among adult PLHIV between Kenya’s 2014 national treatment guidelines and UNAIDS’ 90-90-90 goals.

## Materials and Methods

### Study design and Population

KAIS 2012, described in detail elsewhere, was a population-based household survey of persons aged 18 months to 64 years, conducted from October 2012 to February 2013 [10]. The survey provided representative estimates of HIV prevalence and coverage of HIV prevention, care, and treatment services in nine out of ten geographic regions in Kenya. Briefly, the KAIS 2012 used a two-stage cluster sampling design. In the first stage household clusters were systematically sampled from a national household sampling frame. In the second stage, eligible households were selected using equal probability systematic sampling. Due to regional insecurity, the North Eastern region bordering Somalia was not included in the national sampling frame and thus, not included in the survey.

### Data collection

Individual questionnaires were administered to consenting participants by trained interviewers. Questionnaires collected information on demographics, HIV testing history, HIV status, and ART status among persons who self-reported HIV-positive status. Survey phlebotomists collected whole blood samples from participants. Blood samples were transported to the national HIV reference laboratory for HIV testing, and, if HIV-positive, additional testing for CD4+ T-cell counts and HIV-1 RNA concentration. Participants were informed that results from laboratory testing would not be returned to them but were offered to learn their HIV status immediately using home-based testing and counselling.

### Laboratory methods

Specimens were screened for HIV antibodies using the Vironostika HIV Uni-Form 2012 Plus O Enzyme Immunoassay (bioMerieux, Marcy D’Etoile, France) and confirmed using the Murex HIV.1.2.O HIV Enzyme Immunoassay (DiaSorin, SpA, Saluggia, Italy). Specimens that were not reactive on the screening assay were classified as HIV-negative. Specimens that were reactive on the screening and confirmatory assay were classified as HIV-positive. Specimens with discordant results after confirmatory testing were re-tested using the same algorithm above. Specimens with dually discordant results were tested with polymerase chain reaction (Cobas Amplicor HIV-1 Monitor Test, version 1.5, Roche Molecular Diagnostics, Pleasanton, CA), and these results were used as the final HIV result. HIV-positive samples were further tested for CD4+ T-cell counts using BD FACSCalibur flow cytometer (Becton Dickinson BioSciences, San Jose, CA) and HIV-1 RNA concentration (Abbott M2000 Real-Time HIV-1 Assay, Abbott Laboratories, Abbott Park, IL). Any blood samples remaining after HIV, CD4, and viral load testing were stored at -70°C for future testing. Stored samples were tested for the presence of antiretroviral (ARV) drugs approximately two years after the end of data collection using a qualitative ARV drug assay that tested for nevirapine, efavirenz, lamivudine, and lopinavir using liquid chromatography-tandem mass spectrometry. Specimens that tested positive for one or more ARV drugs were classified as having biological confirmation of ART.

### Measures

Respondents were classified as PLHIV if they had an HIV-positive test based on laboratory results from the survey. PLHIV were classified as having diagnosed HIV infection if they self-reported HIV-positive status during the interview or had biological confirmation of ART. PLHIV were classified as being on ART if they had biological confirmation of ART or self-reported ART use if ARV drug test results were not available. Eligibility criteria for ART initiation were based on the 2014 Kenyan HIV treatment guidelines which recommended ART for HIV-infected persons aged 15 years and older with CD4 ≤ 500 cells/mm^3^ or, irrespective of CD4 counts, HIV-infected persons with active tuberculosis disease, chronic Hepatitis B virus (HBV) infection requiring treatment, HIV-infected women who were pregnant or lactating, or HIV-infected partners in HIV serodiscordant partnerships. Because persons who were currently on ART would continue to need treatment in the future, they were also included in the ART-eligible population. Self-reported information on current tuberculosis treatment was used as a proxy for active tuberculosis disease. Clinical staging and HBV infection status was not collected in the survey and therefore not included in the eligibility criteria in this analysis. The 90-90-90 targets were defined as: 1) 90% of PLHIV diagnosed; 2) 90% of diagnosed PLHIV on ART; and 3) 90% of PLHIV on ART with viral suppression.^3^ PLHIV were classified as virally suppressed if their HIV-1 RNA concentration was under 1,000 copies per milliliter.

### Data Analysis

We limited our analysis to adults and adolescents aged 15–64 years with survey results available from CD4+ T-cell count testing at the central laboratory. This age group represented a sub-population that covered 55% of the total population and 92% of all PLHIV in 2012. Fifty-four percent of HIV-infected respondents had randomly missing CD4+ T-cell count test results due to hemolysis of blood samples that occurred during specimen transport to the central laboratory. We modified the survey’s blood weights to account for missing CD4+ T-cell count data for HIV-infected participants and applied these modified weights to all variables that included biomarkers in its construction, including HIV infection, knowledge of HIV-positive status, ART eligibility, ART coverage, and viral suppression. Data analyses were performed in SAS version 9.3 (SAS Institute, Inc., Cary, NC) using SURVEYFREQ procedures to adjust for survey design, sampling probability, and nonresponse.

Coverage of HIV diagnosis was calculated for two populations in need of HIV testing based on national and UNAIDS targets: 1) persons eligible for ART initiation based on the 2014 Kenyan ART guidelines and 2) all PLHIV. Coverage for ART and viral suppression was calculated for three populations in need of viral suppression based on national and UNAIDS targets: 1) persons eligible for ART initiation based on the 2014 Kenyan ART guidelines; 2) diagnosed PLHIV; and 3) all PLHIV. Unmet need for HIV diagnosis, ART, and viral suppression was calculated by subtracting the number of PLHIV with positive responses in these three strata from the total number of persons in need of HIV diagnosis, ART, and viral suppression, respectively. We applied non-normalized survey weights, based on the 2012 projected population estimates in the 2009 Kenya Population and Housing Census, to estimate population sizes for UNAIDS’ 90-90-90 targets, ART eligibility, and coverage and unmet need for HIV diagnosis, ART, and viral suppression [11]. In this analysis, we present estimates for Kenya and Nyanza region separately.

### Ethical approval

The KAIS 2012 protocol was approved by the Ethical Review Board of the Kenya Medical Research Institute, the Institutional Review Board of the US Centers for Disease Control and Prevention, and the Committee on Human Research of the University of California, San Francisco. For consistency with previous national household surveys in Kenya, a waiver of written documentation of informed consent was requested and approved by the three ethical review entities. Verbal consent was obtained from participating adults aged 18–64 years and parents or guardians of participating minors aged 18 months to 17 years and documented with a signature from study staff. A second signature of study staff was provided for documenting verbal assent from participating minors aged 10–17 years.

## Results

In 2012, there were an estimated 1,147,000 (95% CI: 1,014,000 to 1,279,000) persons aged 15–64 years living with HIV in Kenya, and over one-third (35.5%) or 407,000 PLHIV (95% CI: 317,000 to 497,000) resided in Nyanza (Table 1). For UNAIDS’ 90-90-90 targets to be reached in 2012, 1,032,000 PLHIV (95% CI: 913,000 to 1,151,000) nationally and 366,000 PLHIV (95% CI: 285,000 to 447,000) in Nyanza required diagnosis; 929,000 PLHIV (95% CI: 821,000 to 1,036,000) nationally and 330,000 PLHIV (95% CI: 257,000 to 403,000) in Nyanza required treatment; and 836,000 PLHIV (95% CI: 739,000 to 932,000) nationally and 297,000 PLHIV (95% CI: 231,000 to 362,000) in Nyanza required viral suppression. Nationally, 79.0% (95% CI: 74.2 to 83.7) of PLHIV or 905,000 persons (95% CI: 783,000 to 1,027,000) were eligible for ART based on criteria outlined in Kenya’s 2014 ART guidelines (Table 2). In Nyanza, 80.6% (95% CI: 75.1 to 86.1) of PLHIV or 329,000 persons (95% CI: 253,000 to 404,000) were eligible for ART under these guidelines.

**Table 1:** Joint United Nations Programme on HIV/AIDS 90-90-90 targets in Kenya and Nyanza region, Kenya AIDS Indicator Survey, 2012–2013.

Indicator	Target	Kenya	Nyanza region
		N (range)	N (range)
All PLHIV	100%	1,147,000 (1,014,000, 1,279,000)	407,000 (317,000, 497,000)
Diagnosed	90%	1,032,000 (913,000, 1,151,000)	366,000 (285,000, 447,000)
Diagnosed, on ART	90%	929,000 (821,000, 1,036,000)	330,000 (257,000, 403,000)
Diagnosed, on ART, virally suppressed	90%	836,000 (739,000, 932,000)	297,000 (231,000, 362,000)

**Table 2:** Coverage of diagnosis, treatment, and viral suppression among PLHIV eligible for treatment in Kenya and Nyanza region based on 2014 national treatment guidelines, Kenya AIDS Indicator Survey, 2012–2013.

	Kenya		Nyanza region	
	% (95% CI)	N (range)	% (95% CI)	N (range)
PLHIV	100%	1,147,000 (1,140,000, 1,279,000)	100%	407,000 (317,000, 497,000)
ART-eligible^†^	79.0% (74.2, 83.7)	905,000 (783,000, 1,027,000)	80.6% (75.1, 86.1)	329,000 (253,000, 404,000)
Coverage of diagnosis among ART-eligible	71.3% (64.3, 78.3)	645,000 (530,000, 761,000)	68.3% (55.1, 81.5)	224,000 (157,000, 292,000)
Coverage of ART among ART-eligible	56.9% (49.3, 64.4)	515,000 (412,00, 617,000)	47.7% (35.3, 60.1)	157,000 (101,000, 212,000)
Coverage of ART among ART-eligible and diagnosed	79.8% (72.8, 86.8)	515,000 (412,000, 617,000)	69.8% (57.0, 82.6)	157,000 (101,000, 212,000)
Coverage of viral suppression among ART-eligible	51.8% (43.5, 60.0)	460,000 (353,000, 567,000)	44.1% (31.3, 56.8)	141,000 (85,000, 199,000)
Coverage of viral suppression among ART-eligible and on ART	79.8% (71.4, 88.3)	408,000 (307,000, 509,000)	78.7% (66.2, 91.2)	122,000 (72,000, 172,000)

^†^ Under the 2014 Kenya national ART guidelines, ART eligibility included all persons currently on ART and persons not on ART who had CD4 ≤ 500 cells/mm^3^, women who were pregnant or breastfeeding irrespective of CD4+ T-cell count, and HIV-infected partners in a discordant relationship irrespective of CD4+ T-cell count.

### Coverage of HIV diagnosis

Nationally, coverage of HIV diagnosis was 62.4% (95% CI: 55.3 to 69.5) among all PLHIV and 71.3% (95% CI 64.3 to 78.3) among PLHIV eligible for ART (Tables 2 and 3).

**Table 3:** Coverage of diagnosis, treatment, and viral suppression among PLHIV in Kenya and Nyanza region, Kenya AIDS Indicator Survey, 2012–2013.

	Kenya		Nyanza region	
Indicator	% (95% CI)	N (range)	% (95% CI)	N (range)
All PLHIV	100%	1,147,000 (1,014,000, 1,279,000)	100%	407,000 (317,000, 497,000)
Coverage of diagnosis among all PLHIV	62.4% (55.3, 69.5)	716,000 (593,000, 838,000)	60.1% (47.0, 73.1)	245,000 (170,000, 319,000)
Coverage of ART among all PLHIV	44.9% (38.0, 51.8)	515,000 (406,000, 624,000)	38.4% (27.9, 49.0)	157,000 (97,000, 216,000)
Coverage of ART among diagnosed PLHIV	71.9% (64.3, 79.6)	515,000 (406,000, 624,000)	64.0% (50.4, 77.7)	157,000 (97,000, 216,000)
Coverage of viral suppression among all PLHIV	44.5% (37.0, 51.9)	497,000 (389,000, 606,000)	38.3% (27.2, 49.4)	152,000 (92,000, 212,000)
Coverage of viral suppression among PLHIV on ART	79.8% (71.4, 88.2)	408,000 (304,000, 512,000)	78.7% (66.3, 91.1)	122,000 (69,000, 175,000)

In Nyanza, 60.1% (95% CI: 47.0 to 73.1) of PLHIV and 68.3% (95% CI: 55.1 to 81.5) of those eligible for ART had been diagnosed.

**Fig 1:**
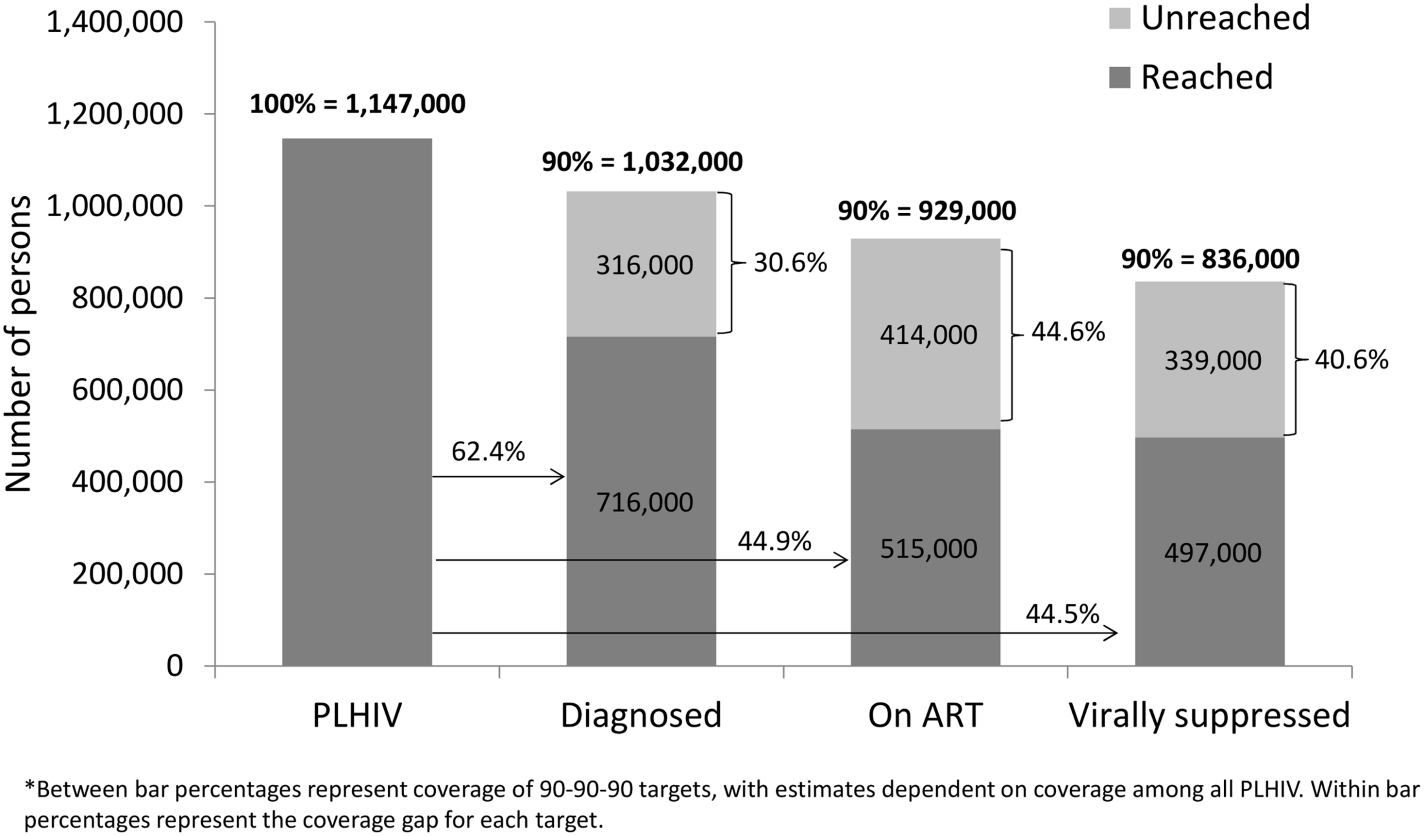
Baseline estimates of coverage of 90-90-90 targets in Kenya, Kenya AIDS Indicator Survey, 2012–2013*.

**Fig 2:**
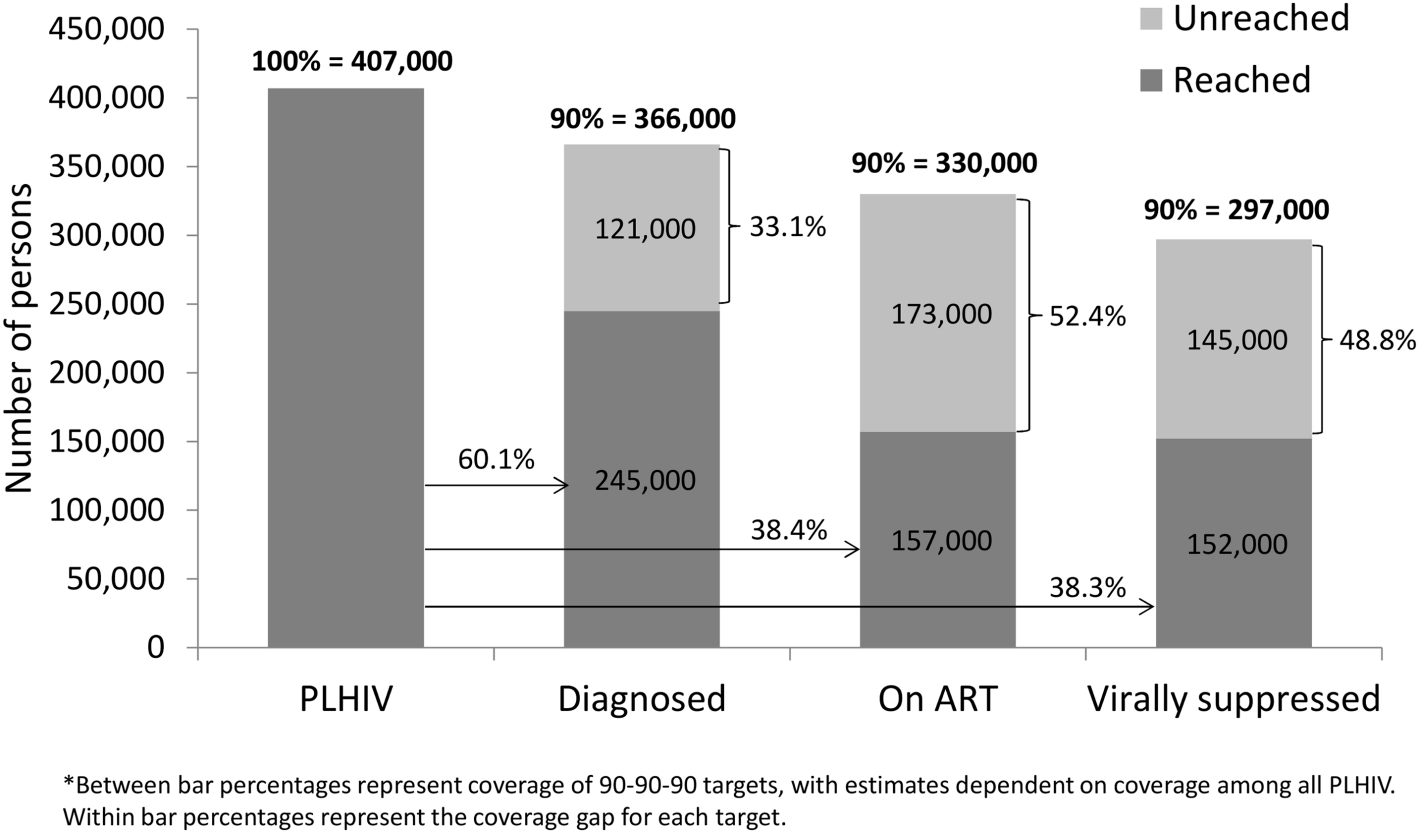
Baseline estimates of coverage of 90-90-90 targets in Nyanza region, Kenya AIDS Indicator Survey, 2012–2013*.

**Fig 3:**
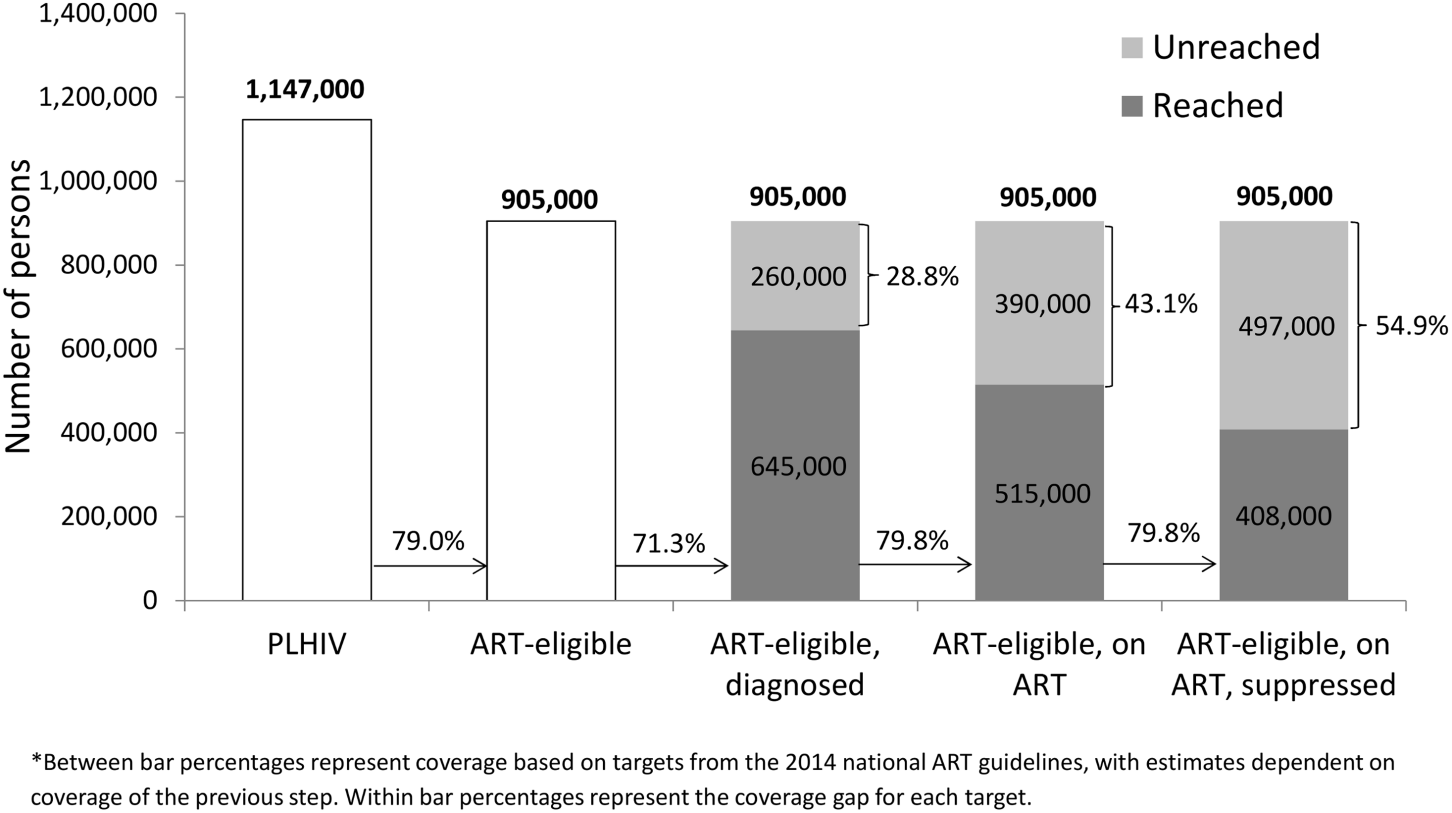
Baseline estimates of coverage of diagnosis, treatment, and viral suppression among persons eligible for ART in Kenya based on the 2014 national treatment guidelines, Kenya AIDS Indicator Survey, 2012–2013*.

**Fig 4:**
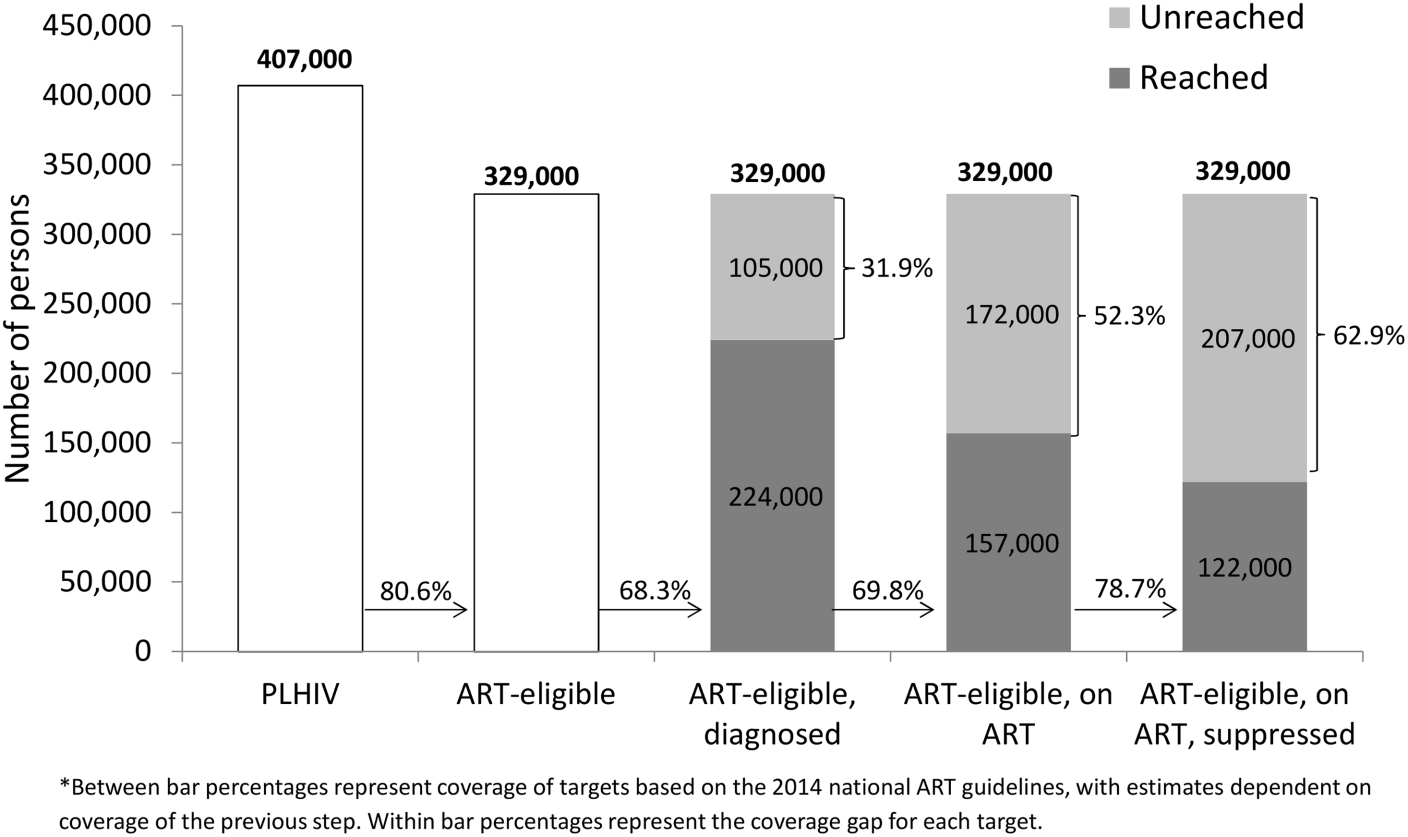
Baseline estimates of coverage of diagnosis, treatment, and viral suppression among persons eligible for ART in Nyanza region based on the 2014 national treatment guidelines, Kenya AIDS Indicator Survey, 2012–2013*.

To meet the unmet need for HIV diagnosis based on UNAIDS’ first “90” (i.e., 90% of PLHIV diagnosed), an additional 316,000 persons (30.6% of target) would need to be diagnosed nationally and an additional 121,000 persons (33.1% of target) would need to be diagnosed in Nyanza (Figs 1 and 2). In comparison, the unmet need for HIV diagnosis among all ART-eligible PLHIV in 2012 was lower at the national level, with an additional 260,000 persons (28.8% of target) who needed to be diagnosed but similar in Nyanza, with an additional 105,000 persons (31.9%) who required diagnoses (Figs 3 and 4).

Nationally, the need for diagnosis based on Kenya’s treatment guidelines (n = 905,000) was 87.7% of need for diagnosis based on UNAIDS’ 90-90-90 targets (n = 1,032,000). In Nyanza, the need for diagnosis based on the national treatment guidelines (n = 329,000) was 89.9% of need for diagnosis based on UNAIDS’ 90-90-90 targets (n = 366,000). Based on this an additional 127,000 persons nationally and 37,000 persons in Nyanza who would be targeted for diagnosis under the UNAIDS strategy would be left unaccounted for under Kenya’s current treatment policy.

### Coverage of treatment

Among all PLHIV, 515,000 persons (95% CI: 406,000 to 624,000) were on ART, representing a coverage of 44.9% (95% CI: 38.0 to 51.8) among all PLHIV, 56.9% (95% CI: 49.3 to 64.4) among those eligible for ART, and 71.9% (95% CI: 64.3 to 79.6) among those diagnosed (Tables 2 and 3). In Nyanza, 157,000 persons (95% CI: 97,000 to 216,000) were currently on ART, covering 38.4% (95% CI: 27.9 to 49.0) of all PLHIV, 47.7% (95% CI: 35.3 to 60.1) of those eligible for ART, and 64.0% (95% CI: 50.4 to 77.7) of those already diagnosed.

Given this level of treatment coverage, resolving the unmet need for treatment based on UNAIDS’ second “90” (i.e., 90% of diagnosed PLHIV on ART) would require placing an additional 414,000 persons (44.6% of target) nationally and 173,000 persons (52.4% of target) in Nyanza on ART (Figs 1 and 2). Comparatively, the unmet need for treatment among those eligible for ART based on Kenya’s ART guidelines would require an additional 390,000 persons (43.1% of target) nationally and 172,000 persons (52.3% of target) in Nyanza to access ART (Figs 3 and 4).

Total need for treatment based on Kenya’s ART guidelines (n = 905,000) was 97.4% of treatment need based on UNAIDS’ 90-90-90 treatment targets (n = 929,000). This means that an additional 24,000 PLHIV beyond those eligible for ART still needed treatment to achieve the 90-90-90 treatment target. In Nyanza, the target for ART need based on treatment eligibility (n = 329,000) was nearly equivalent to the 90-90-90 treatment target for Nyanza (n = 330,000).

### Coverage of viral suppression

Nationally, 497,000 PLHIV (95% CI: 389,000 to 606,000) were virally suppressed in 2012, representing 44.5% (95% CI: 37.0 to 51.9) of all PLHIV and 79.8% (95% CI: 71.4 to 88.2) of all PLHIV on ART (Table 2). Among PLHIV eligible for ART, 460,000 persons (95% CI: 353,000 to 567,000) were virally suppressed, covering 51.8% (95% CI: 43.5 to 60.0) of treatment-eligible persons (Table 2). In Nyanza, 152,000 persons (95% CI 92,000 to 212,000) had achieved viral suppression, covering 38.3% (95% CI: 27.2 to 49.4) of all PLHIV and 78.7% (95% CI: 66.3 to 91.1) of PLHIV on ART. Among persons eligible for treatment in Nyanza, 141,000 persons (95% CI: 85,000 to 199,000) were virally suppressed, representing 44.1% (95% CI: 31.3 to 56.8) of treatment-eligible PLHIV in the region.

To address the unmet need for viral suppression based on UNAIDS’ third “90” (i.e., 90% of PLHIV on ART with viral suppression) an additional 339,000 persons (40.6% of target) would need to be suppressed nationally and an additional 145,000 persons (48.8% of target) would need to be suppressed in Nyanza (Figs 1 and 2). The unmet need for viral suppression based on Kenya’s ART guidelines was higher, requiring an additional 497,000 persons (54.9% of target) nationally and an additional 207,000 persons (62.9% of target) in Nyanza to be suppressed (Figs 3 and 4).

Nationally, the need for viral suppression based on Kenya’s national treatment guidelines (n = 905,000) was 108% the need for viral suppression based on UNAIDS’ third “90” (n = 836,000). In Nyanza, the need for viral suppression based on the national ART guidelines (n = 329,000) was 111% the need for viral suppression based on UNAIDS’ third “90” (n = 297,000).

## Discussion

In 2012, approximately eight in ten adult and adolescent PLHIV in Kenya needed treatment based on eligibility criteria outlined in the 2014 national ART guidelines. A large proportion of individuals requiring ART were from Nyanza, where ART coverage among all PLHIV and PLHIV eligible for ART was seven and nine percentage points lower, respectively, than the country as a whole. Though there were some differences in estimates of need and unmet need for diagnosis, treatment, and viral suppression between the Kenya’s national ART guidelines and UNAIDS’ 90-90-90 goals, they were still fairly similar. These findings suggest that if Kenya is able to identify and treat all persons eligible for ART under its current national treatment policy, UNAIDS’ 90-90-90 treatment targets can be achieved in the high burden region of Nyanza but would fall shy of the national target by approximately 24,000 PLHIV.

In spite of these minor differences, there is still a considerable amount of work needed to address the unmet need for treatment in the country, particularly in Nyanza where five in ten treatment-eligible persons had not yet accessed ART by 2012. The alignment of Kenya’s national treatment guidelines with the 2015 WHO recommendations to treat all PLHIV irrespective of immunologic status will play a critical role in accelerating access to ART to those who need it [4]. However this cannot be realized without the substantial scale-up of first “90”—greatly increased knowledge of HIV status through parallel scale-up of HIV testing. Based on routine programmatic monitoring data reported in 2012, the percentage of persons that tested HIV-positive through community and health facility testing was 4.5% (unpublished work). Considering that the level of undiagnosed infection in the adult population is approximately 2% [5], in order for 90% of PLHIV to know their status, an estimated 11 million persons would need to be tested. This represents over half of adults and adolescents aged 15–64 years and three million more additional HIV tests than those conducted in 2012.

To achieve universal knowledge of serostatus, innovative strategies targeted to geographic settings and populations with heaviest HIV burden are needed [12]. Testing family members of HIV-positive persons presents a clear avenue for identifying potentially large numbers of HIV-infected persons that can be linked immediately to care. Given that one-third of persons with tuberculosis disease are also living with HIV disease, HIV testing of TB patients also presents an enormous opportunity to identify a large numbers of persons with unmet need for treatment [13]. If 95% of TB and HIV co-infected cases are identified and all receive ART, it is expected that TB patients could contribute to 10% of the unreached population [8]. Additionally, by increasing testing coverage to 95% of all pregnant women accessing antenatal care and linking 95% of those HIV-positive into care, it is expected that pregnant women could account for 15% of persons with unmet need for ART [8]. Although provider-initiated testing and counselling has the potential to test high volumes of persons seeking health care services, resources for scale-up should be prioritized in facilities located in high prevalence settings to maximize yield. Community-based testing strategies, such as home-based testing and counselling, national testing campaigns, and self-testing, delivered in settings with high HIV burden, are also needed to identify undiagnosed infection in healthy populations that would otherwise not seek care [14].

Though important progress has been made in treatment access since 2012, ART coverage among adult PLHIV was still low in 2014 with only six in ten receiving ART by the end of the year [8]. The success of the second “90” can only be achieved by adapting aggressive approaches to rapidly resolve the large treatment gap. Mathematical models have demonstrated that universal test and treat (UTT) at a coverage rate of 90% can eliminate HIV disease within 20–25 years [15,16]. For UTT to succeed, it will require intensified and repeated testing to identify PLHIV, immediate and sustained engagement in HIV care for diagnosed PLHIV, and the ability of the health care system to rapidly absorb and treat high numbers of PLHIV. The efficacy of UTT is being evaluated in several large-scale community trials, including the Strategic Timing of AntiRetroviral Treatment (START) study [17–22]. In 2015, START found that starting ART early at CD4 > 500 cells/mm^3^ reduced the risk of HIV morbidity and mortality by more than 50% compared to starting treatment later at CD4 ≤ 350 cells/mm^3^ [22]. As it is unlikely that UTT alone will be enough to end the epidemic, combination approaches using a mix of prevention strategies, including UTT, voluntary medical male circumcision, and condoms, delivered in areas and populations with greatest need, is still the most effective approach to reverse the epidemic [12].

Although the level of undetectable virus among treated PLHIV was concomitant with levels observed in developed settings, only four in ten adult and adolescent PLHIV were virally suppressed [9]. The success of the third “90” will be dependent on optimal coverage of each component of the care continuum: the ability for PLHIV to be diagnosed, linked to care, remain in care, initiate ART, and adhere to their medication. Programmatic data highlight encouraging progress but sobering gaps in each of the steps of the continuum of care. Retention at 12 months is 80%, viral suppression at 12–36 months on treatment is 89%, and prevalence of drug resistance mutations among those in care and failing treatment is 92% (personal communication, National AIDS and STI Control Programme, Ministry of Health, Kenya, May 29, 2015).

As treatment is rapidly delivered to those who need it, the interventions needed to address the gaps at each step in the treatment cascade will become even more challenging as more people are diagnosed and linked into care. Regular monitoring of emergence and transmission of HIV drug resistance will be critical to ensure that intensified treatment efforts are not undermined. Moreover, the impact of these interventions on viral suppression at the population level cannot be verified without routine, high-quality data on the status of the treatment cascade. HIV case-based surveillance, which follows cohorts of HIV-positive individuals from diagnosis through sentinel events in disease progression, will provide important information to monitor the cascade at both national and sub-national levels [23]. However, until these systems improve in SSA, population-based surveys continue to serve as an accurate representation of the clinical cascade for a country.

This analysis cannot be interpreted without consideration of the following limitations. Because the North Eastern region was not included in the survey, the national estimates presented may not be representative of the country as a whole. However, based on the last two national HIV serologic surveys conducted in Kenya, North Eastern region had the lowest HIV prevalence in the country (0.8% in 2007 and 0.9% in 2008/2009) [24,25]. As such, the exclusion of North Eastern region in the survey is expected to have limited impact on the generalizability of our findings. Over fifty percent of HIV-positive survey respondents had randomly missing CD4+ T-cell count test results due to hemolysis of blood that occurred during specimen transport. No differences in sex, age, residence, and geographic region were observed among persons with and without CD4+ T-cell count data. Therefore any potential bias from this loss is expected to be minimal. Because our analysis excluded respondents that did not have a CD4+ T-cell count test result, the smaller sample size resulted in higher coverage estimates of 90-90-90 targets with wider confidence bounds compared to corresponding 90-90-90 coverage estimates based on the full sample of HIV-infected respondents. In spite of this, the differences were not statistically significant: 62.4% (95% CI: 55.3 to 69.5) vs. 56.2% (95% CI: 50.7 to 61.7) for HIV diagnosis, 44.9% (95% CI: 38.0 to 51.8) vs. 42.5% (95% CI: 37.4 to 47.4) for treatment, and 44.5% (95% CI: 37.0 to 51.9) vs. 40.4% (95% CI: 35.5 to 45.3) for viral suppression, respectively. Though we strengthened our analysis by using biological confirmation of ART to identify persons aware of their HIV infection and on ART, 14% of HIV-positive specimens had ARV drug test results missing at random due to insufficient samples. Because we relied on self-reported information to determine final ART status for respondents with missing ARV drug test results, it possible that some individuals may have misreported their true ART status. Additionally, because presence of ARV drugs in the blood is a measure of recent use, individuals who were not adhering to their medication may have tested negative for ART reflecting an underestimation of ART use in this analysis. In our sample, 5% of specimens that did not have presence of ARV drugs in their blood were from HIV-infected respondents who had self-reported ART use [26]. Moreover, because the survey did not collect information on chronic HBV infection or disease staging, these criteria were not included in our definition of ART eligibility. However, the proportion of patients with CD4 > 500 cells/mm^3^ who were newly enrolled in care with chronic HBV infection or stage 3 or 4 disease is expected to be very low (unpublished work). Nonetheless, ART coverage may be underestimated while unmet need for treatment may be overestimated. It is also possible that we underestimated the level of diagnosed infection as some persons in care, but not on ART may have under-reported their HIV-positive status. Finally, since 2012, the 2014 national ART guidelines and 90-90-90 treatment strategies have been implemented in Kenya, resulting in increases in coverage of HIV diagnosis and treatment. The findings of this analysis, therefore, should only be considered baseline estimates of coverage prior to adoption of these strategies.

Despite these limitations, the strength of this analysis is the fairly representative sample that was used to improve our knowledge of population-level need and coverage of HIV diagnosis, treatment, and viral suppression among adult and adolescent PLHIV in Kenya and in Nyanza, the region most heavily burdened by HIV disease in the country. These findings serve as important benchmarks for measuring progress towards treatment targets set through Kenya’s current national treatment policy, UNAIDS’ 90-90-90 goals, and 2015 WHO treatment recommendations to treat all PLHIV regardless of CD4+ T-cell count status. Continuing the pace at which treatment scale-up is occurring in Kenya will result in thousands of PLHIV unreached, the majority of whom will have detectable viral load and at risk of transmitting infection to others. Providing treatment, irrespective of eligibility, has the potential to reach all who require ART but will necessitate a careful shift in resources to prioritize geographic settings with the highest numbers of HIV infections and new implementation strategies to accelerate coverage to where it is most needed [12]. Thoughtful discourse with the Government of Kenya and key stakeholders on best strategies for implementing universal treatment for all PLHIV to achieve 90-90-90 goals will be instrumental in determining whether intensified access to treatment can be achieved.
